# Polystyrene Microplastics Impair Intestinal Homeostasis in Juvenile *Tachypleus tridentatus* Through Oxidative Imbalance and Gut Microbiota Dysregulation

**DOI:** 10.3390/toxics14090797

**Published:** 2026-09-09

**Authors:** Yuhong Li, Yiran Tan, Liyun Han, Yue Liu, Mingxiao Liu, Caoqun Zheng, Wenxin Jin, Tianshuai Zhang, Bosen Weng, Zhaohong Weng, Jun Bo

**Affiliations:** 1 Department of Environmental Science and Engineering, College of Chemical Engineering, Huaqiao University, Xiamen 361021, China; 2 Fujian Provincial Key Laboratory of Marine Fishery Resources and Eco-Environment, Fisheries College of Jimei University, Xiamen 361021, China; 3 Key Laboratory of Marine Ecological Conservation and Restoration, Third Institute of Oceanography, Ministry of Natural Resources, Xiamen 361005, China

**Keywords:** *Tachypleus tridentatus*, polystyrene microplastics, intestinal toxicity, oxidative stress, gut microbiota, ecological risk

## Abstract

Microplastics (MPs) are ubiquitous marine pollutants that pose increasing ecological risks, yet their effects on intestinal physiology and gut microbial homeostasis during the juvenile developmental stages of *Tachypleus tridentatus* remain unclear. In this study, fifth- and sixth-instar juveniles of *T. tridentatus* were exposed to environmentally relevant concentrations of 6.0 μm polystyrene microplastics (PS-MPs; 0, 10^2^, and 10^4^ particles/L) for 7 and 21 days to investigate intestinal physiological responses and potential mechanisms associated with PS-MP exposure. Intestinal retention, oxidative stress, innate immune responses, and gut microbiota were comprehensively evaluated. PS-MP exposure induced concentration-dependent and time-dependent alterations in superoxide dismutase, catalase, malondialdehyde, and lysozyme, suggesting oxidative imbalance and modulation of innate immune responses. Fifth-instar juveniles displayed more pronounced oxidative-stress alterations and divergent innate-immune profiles relative to sixth-instar conspecifics, pointing to greater physiological susceptibility at earlier developmental stages. Gut microbiota analysis revealed pronounced dysbiosis, characterized by a reduced relative abundance of Bacillota, enrichment of Pseudomonadota, depletion of beneficial taxa (e.g., *Lactococcus*), and increased abundance of opportunistic bacteria, including *Pseudomonas* and members of Enterobacteriaceae. These physiological and microbial alterations collectively suggest that environmentally relevant PS-MPs impair intestinal homeostasis in juvenile *T. tridentatus* by inducing oxidative imbalance, modifying innate immune responses, and reshaping gut microbial communities, with clear instar- and exposure time-dependent effects. These findings highlight the importance of considering developmental stages in ecological risk assessment for benthic arthropods.

## 1. Introduction

*Tachypleus tridentatus* (Leach, 1819) (Chinese horseshoe crab) is an ancient marine chelicerate and one of only four extant horseshoe crab species worldwide. Owing to its highly conserved morphology over more than 400 million years of evolution, it is widely regarded as a “living fossil” and has considerable value in evolutionary biology, comparative physiology, and biomedicine, particularly as the natural source of limulus amebocyte lysate (LAL) reagents [1]. Despite its ecological and biomedical importance, wild populations of *T. tridentatus* have experienced severe declines in recent decades because of habitat degradation, coastal development, and overexploitation, resulting in its classification as an endangered species [2]. Juvenile horseshoe crabs primarily inhabit estuarine, intertidal, and shallow coastal environments, where they remain closely associated with sediments during burrowing and feeding activities. These habitats are recognized as major sinks for microplastics, exposing juvenile horseshoe crabs to persistent sediment-associated contaminants throughout their early development [1,3]. Recent studies have indicated that microplastic exposure adversely affects growth, behaviour, and physiological performance in juvenile *T. tridentatus*, while early developmental stages appear particularly susceptible to contaminant-induced oxidative stress [4,5]. Collectively, these characteristics highlight *T. tridentatus* as an ecologically relevant model for evaluating the biological effects of microplastic pollution in coastal benthic ecosystems.

Among the diverse environmental pressures affecting coastal ecosystems, microplastic pollution has emerged as a pervasive threat to aquatic organisms. Microplastics (MPs) are defined as particles with a maximum axial dimension ranging from 1 μm to 5 mm [6], which are ubiquitous contaminants detected globally in marine waters, estuaries and sediments [7]. Field-measured microplastic abundances in many coastal waters can reach levels on the order of 10^2^ particles/L [7,8,9,10], while the higher concentration of 10^4^ particles/L represents a moderate-stress experimental exposure scenario frequently adopted in laboratory ecotoxicity assays [11]. Polystyrene microplastics (PS-MPs), one of the most frequently detected polymer types in aquatic environments [12], exhibit high environmental persistence and physicochemical stability, enabling long-distance translocation through hydrological processes and trophic transfer within aquatic food webs [13,14,15,16]. Consequently, PS-MPs have become an important focus of ecotoxicological research because of their widespread occurrence and increasing ecological risks to marine organisms. Although previous studies have documented adverse effects of MPs on growth, behaviour, and reproduction across diverse aquatic taxa [17], their impacts on intestinal physiology and host–microbiota interactions remain incompletely understood, especially for marine benthic arthropods [18].

Accumulating evidence indicates that the intestine is one of the primary target organs of microplastic toxicity in aquatic organisms [19,20]. In addition to causing physical abrasion and obstruction within the digestive tract, MPs can induce excessive production of reactive oxygen species (ROS), leading to oxidative stress, impairment of antioxidant defense systems, and impairment of innate immune function [21]. As the largest interface between the host and the external environment, the intestine harbors complex microbial communities that are essential for nutrient metabolism, immune regulation, and maintenance of intestinal barrier integrity [22,23]. Disturbance of gut microbial homeostasis is frequently characterized by the depletion of beneficial microorganisms and enrichment of opportunistic pathogens, thereby exacerbating intestinal inflammation and oxidative damage and ultimately compromising intestinal homeostasis [24,25,26]. Consistent with this mechanism, microplastic exposure has been shown to induce intestinal inflammation accompanied by gut microbial dysbiosis in zebrafish [27]. However, most previous studies have focused on teleost fishes and adult organisms, whereas the intestinal responses of marine benthic arthropods, particularly during juvenile development, remain largely unexplored [28,29]. Six-micrometre polystyrene microplastics represent a widely adopted experimental model: they are readily ingested by animals, predominantly retained within the gastrointestinal lumen, and exhibit limited transepithelial translocation, rendering them suitable for gut-centered toxicity assessments. While prior work has demonstrated that microplastics can trigger antioxidant stress in juvenile horseshoe crabs, no published studies have employed monodisperse spherical 6 μm polystyrene microplastics to explore instar-specific ecotoxicological characteristics in juvenile *T. tridentatus.* For these reasons, we selected 6 μm polystyrene microplastics for the present study [11,20,30]. Consequently, the mechanisms by which environmentally relevant 6 μm PS-MPs alter intestinal physiology and gut microbial homeostasis in juvenile horseshoe crabs remain incompletely resolved. To date, a notable knowledge gap persists regarding instar-specific shifts in intestinal physiology and gut-microbiota profiles of juvenile *T. tridentatus* under environmentally relevant microplastic concentrations. Accordingly, this study integrates measurements of intestinal particle retention, oxidative-stress status, immune biomarkers, and gut-microbiome profiling to dissect instar-dependent intestinal toxicity triggered by PS-MPs.

Juvenile development represents a particularly vulnerable life-history stage in horseshoe crabs because of their Type III survivorship strategy, characterized by high fecundity but extremely low juvenile survival. The 5th- and 6th-instar stages encompass rapid somatic growth and physiological differentiation, during which the intestine plays a pivotal role in nutrient assimilation, immune maturation, and maintenance of host health. Although the gut microbiota of wild juvenile *T. tridentatus* has been preliminarily characterized [31], and several studies have evaluated antioxidant responses to nanoplastics and heavy metals in horseshoe crabs [32,33], important knowledge gaps remain. In particular, few studies have systematically investigated the accumulation of environmentally relevant PS-MPs within the gastrointestinal tract together with concurrent alterations in intestinal antioxidant capacity, innate immune responses, and gut microbial communities. Furthermore, whether susceptibility to microplastic exposure differs between juvenile developmental stages remains unknown. Addressing these knowledge gaps is essential for improving our understanding of the physiological vulnerability of endangered horseshoe crabs to emerging contaminants and for strengthening ecological risk assessments in coastal benthic ecosystems.

Horseshoe crabs possess specialized physiological features, including hemolymph-based innate immunity and are benthic organisms closely associated with sediment-rich estuarine habitats [31]. Their close interaction with coastal sediments, where microplastics are increasingly accumulated, may result in unique exposure pathways and toxicological responses compared with pelagic organisms. Therefore, investigating microplastic-induced intestinal alterations in juvenile horseshoe crabs provides valuable insights into contaminant sensitivity in understudied marine invertebrate taxa.

Therefore, the present study exposed 5th- and 6th-instar juvenile *T. tridentatus* to environmentally relevant concentrations of polystyrene microplastics (0, 10^2^, and 10^4^ particles/L) for 7 and 21 days. The objectives were to (i) characterize the transient occurrence of PS-MPs within the intestine, (ii) evaluate intestinal retention together with changes in antioxidant and innate immune responses, and (iii) determine shifts in gut microbial community composition using 16S rRNA gene sequencing. We hypothesized that environmentally relevant PS-MPs disrupt intestinal homeostasis in juvenile *T. tridentatus* by inducing oxidative stress, impairing innate immune function, and altering gut microbial communities, and that these responses vary with developmental stage and exposure duration. By integrating physiological and microbial endpoints, this study provides a comprehensive evaluation of intestinal toxicity induced by PS-MPs in an endangered marine arthropod. The findings contribute to a better mechanistic understanding of microplastic toxicity in benthic organisms and provide scientific evidence for ecological risk assessment and conservation of the endangered horseshoe crab *T. tridentatus*.

## 2. Materials and Methods

### 2.1. Polystyrene Microplastics

Fluorescently labeled (Cat No. 24157) and non-labeled (Cat No. 07312) polystyrene microspheres with a nominal diameter of 6 μm were purchased from Polysciences Inc. (Warrington, PA, USA). Both products are supplied as aqueous suspensions stabilized with minimal surfactant, without detectable additional additives according to the manufacturer‘s specifications. The nominal coefficient of variance (CV) for particle diameter is 10% as provided by the manufacturer. The stock suspension of PS microspheres had a concentration of 2.10 × 10^8^ particles/mL.

The stock solution was thoroughly homogenized using a vortex mixer and subsequently diluted with distilled water to prepare working stock solutions at concentrations of 2.10 × 10^4^ particles/mL and 2.10 × 10^6^ particles/mL, respectively. All stock solutions were stored at 4 °C until further use. Prior to exposure experiments, the stock suspensions were vortexed again to ensure uniform dispersion, and then added to filtered seawater to prepare the desired exposure concentrations.

Fluorescently labeled PS-MPs were used for particle tracing experiments to observe and enumerate microplastic particles in intestinal tissues via fluorescence microscopy, whereas non-fluorescent PS-MPs were used in all toxicity exposure experiments for the assessment of physiological, biochemical, and molecular endpoints [34,35]. Both types of microspheres share the same polystyrene base material, nominal diameter (6 μm), and theoretical density as supplied by the manufacturer. However, the fluorescently labeled version was prepared by embedding a fluorescent dye within the polymer matrix, and thus the two particle types are not entirely identical in their physicochemical properties. The incorporation of the fluorescent dye may introduce differences in optical properties, and may also potentially affect particle surface characteristics and aggregation behavior in aqueous media. Considering that the fluorescent dye itself could introduce potential biological interference, we did not use fluorescently labeled particles for toxicity testing; they were used exclusively for in vivo distribution tracing. All toxicological effect assessments were conducted using non-fluorescent PS-MPs. According to the manufacturer, the fluorescent dye is stably embedded within the polymer matrix and exhibits no detectable leaching during exposure [36].

### 2.2. Experimental Animals

A total of 300 healthy 5th- and 6th-instar juvenile *T. tridentatus* were provided by Guangxi Marine Research Co., Ltd. (Beihai, China; Breeding permit No.: (Guangxi) Aquatic Wild Breeding No. 2023-0503007). The molting interval between the 5th and 6th instars is approximately six months. Prior to the experiment, all juveniles were acclimated for 10 days in artificial seawater filtered through a 0.22 μm membrane under laboratory conditions. All individuals were in good physiological condition during acclimation (Figure S1).

Morphometric characteristics of juveniles were as follows: 5th-instar individuals had a body weight of 0.47 ± 0.07 g, body length of 3.38 ± 0.41 cm, caudal spine length of 1.64 ± 0.18 cm, prosomal width of 1.96 ± 0.10 cm, and opisthosomal width of 1.36 ± 0.12 cm. For 6th-instar individuals, body weight was 1.28 ± 0.22 g, body length 5.02 ± 0.35 cm, caudal spine length 2.45 ± 0.28 cm, prosomal width 2.69 ± 0.15 cm, and opisthosomal width 2.02 ± 0.15 cm. The photoperiod was set at 14 h light: 10 h dark. Water temperature was maintained at 25 ± 1 °C, salinity at 28–30, pH at 8.3–8.6, and dissolved oxygen ≥ 6 mg/L. Juveniles were fed daily at 17:00 with newly hatched brine shrimp (*Artemia,* Aquamaster Artemia cysts, Zhangzhou, China), at a ration equivalent to approximately 1–2% of their body weight.

### 2.3. Experimental Design

The concentrations of polystyrene microplastics used in this study were selected based on previously reported levels in coastal marine environments [6,8,9,10]. Stock suspensions were diluted to achieve the final exposure concentrations of 0, 1 × 10^2^ particles/L, and 1 × 10^4^ particles/L, respectively, and added to each experimental tank to prepare PS-MP exposure media. The artificial seawater used for preparing PS-MP suspensions was pre-filtered through GF/A nitrocellulose membrane filters (0.22 μm pore size, Millipore, Billerica, MA, USA) to eliminate background particulate contamination.

Prior to exposure, all juvenile *T. tridentatus* were starved for 24 h to minimize the influence of residual gut contents. Individuals with similar body size, healthy external appearance, and relatively uniform initial body mass were selected and randomly assigned to experimental groups. Both fifth- and sixth-instar juveniles were exposed to control (0 particles/L) and two environmentally relevant concentrations of PS-MPs (1 × 10^2^ particles/L and 1 × 10^4^ particles/L) for two exposure durations (7 days and 21 days), respectively. During the light period, the exposure suspensions were gently stirred every 4 h using a sterile glass rod to maintain homogeneous distribution of PS-MPs in the water column. The exposure media were completely renewed every two days, with continuous aeration maintained throughout the experiment. Husbandry conditions during exposure were consistent with those used during acclimation. Each tank contained 15 L of exposure medium containing PS-MPs. The two instars were exposed separately: each 5th-instar and 6th-instar treatment group consisted of 25 individuals per tank (Figure S1).

All juveniles were randomly assigned to six treatment groups according to exposure duration and concentration. For the 7-day exposure groups (R series, R1–R6): R1–R3 represented 6th-instar juveniles, including the control group (no PS-MPs), the low concentration group (10^2^ particles/L), and the high concentration group (10^4^ particles/L), respectively. R4–R6 represented 5th-instar juveniles under the same exposure conditions. Each R group contained five biological replicates, with sample identifiers as follows: R1 (SC1–SC5), R2 (SL1–SL5), R3 (SH1–SH5), R4 (FC1–FC5), R5 (FL1–FL5), and R6 (FH1–FH5). For the 21-day exposure groups (G series, G1–G6): G1–G3 represented 6th-instar juveniles, including control (no PS-MPs), low concentration (10^2^ particles/L), and high concentration (10^4^ particles/L), respectively. G4–G6 represented 5th-instar juveniles under the same exposure conditions. Each G group also contained five biological replicates, with sample identifiers as follows: G1 (SN1–SN5), G2 (SP1–SP5), G3 (SS1–SS5), G4 (FN1–FN5), G5 (FP1–FP5), and G6 (FS1–FS5).

### 2.4. Fluorescence Microscopic Observation of PS-MP Retention in the Gastrointestinal Tract of Juvenile T. tridentatus

After 7 and 21 days of exposure in fluorescent microplastic suspensions, three individuals of similar body size were randomly selected from each treatment group. The juveniles were anesthetized using 200 mg/L ethyl 3-aminobenzoate methanesulfonate (MS-222, Sigma-Aldrich, St. Louis, MO, USA).

The gastrointestinal tracts were carefully dissected on ice and immediately subjected to observation. The morphology, density, and retention patterns of PS-MPs within the digestive tract were examined using an inverted fluorescence microscope (Axio Observer Z1, Carl Zeiss, Oberkochen, Germany) (10× eyepiece and 10× objective lenses, total magnification: 100×).

### 2.5. Determination of Gastrointestinal Physiological Parameters

After 7 and 21 days of PS-MP exposure, nine juvenile *T. tridentatus* were randomly selected from each treatment group, with three individuals taken from each of three independent culture tanks per treatment group. Every three individuals from the same tank were pooled as one composite sample, resulting in three biological replicates per group (*n* = 3), with each replicate representing an independent culture tank.

The juveniles were anesthetized using 200 mg/L ethyl 3-aminobenzoate methanesulfonate (MS-222), and the gastrointestinal tracts were dissected on ice. The collected tissues were rapidly flash-frozen in liquid nitrogen and stored at −80 °C. All samples were homogenized and processed for crude enzyme extraction within 24 h. A measured amount of intestinal tissue was accurately weighed and homogenized in 600 μL of ice-cold 0.9% physiological saline using a glass homogenizer under ice bath conditions. The homogenates were centrifuged at 2500 rpm for 15 min at 4 °C. The resulting supernatants were aliquoted into 1.5 mL centrifuge tubes and used for subsequent biochemical analyses, including total protein (TP) content, catalase (CAT) activity, superoxide dismutase (SOD) activity, malondialdehyde (MDA) content, and lysozyme (LZM) activity.

All assay kits were purchased from Nanjing Jiancheng Bioengineering Institute (Nanjing, China). The measurements of all physiological parameters were conducted strictly according to the manufacturer’s instructions.

### 2.6. Gastrointestinal Tract Microbiota Composition Analysis

Five juvenile *T. tridentatus* were randomly selected from each treatment group, with individuals collected from three independent culture tanks per treatment group (the five individuals were distributed across the three tanks, with no more than two individuals originating from any single tank), and their gastrointestinal tracts were collected to generate five biological replicates.

Total genomic DNA was extracted from intestinal contents using the TIANamp Stool DNA Kit (Tiangen Biotech, Beijing, China) according to the manufacturer’s instructions. DNA quality and concentration were evaluated by agarose gel electrophoresis, and DNA samples were diluted with sterile nuclease-free water to a final concentration of 1 ng/μL. The V4 hypervariable region of the bacterial 16S rRNA gene was amplified using the universal primer pair 515F (5′-GTGYCAGCMGCCGCGGTAA-3′) and 806R (5′-GGACTACNVGGGTWTCTAAT-3′). PCR reactions were performed in a 30 μL volume containing 15 μL of Phusion^®^ High-Fidelity PCR Master Mix (New England Biolabs, Ipswich, MA, USA), 0.2 μM of each primer, and 10 ng of template DNA. The thermal cycling conditions were: initial denaturation at 98 °C for 1 min; 30 cycles of 98 °C for 10 s, 50 °C for 30 s, and 72 °C for 30 s; and a final extension at 72 °C for 5 min.

Sequencing libraries were constructed using the NEBNext^®^ Ultra™ II DNA Library Prep Kit (Cat No. E7645, New England Biolabs, Ipswich, MA, USA) and sequenced on the Illumina NovaSeq 6000 platform (Illumina, Inc., San Diego, CA, USA) with 250 bp paired-end reads. Raw paired-end reads were merged using FLASH (v1.2.11) and quality filtering using fastp (v0.20.0) with default parameters. Chimeric sequences were detected and removed using Vsearch (v2.15.0) against the SILVA reference database (v138). Sequence denoising was subsequently performed using the DADA2 algorithm [37] as implemented in QIIME2 (v2020.06) with trunc-len = 0 and all other parameters set to default, and ASVs with a total abundance of <5 were filtered out. Representative ASV sequences were taxonomically assigned against the SILVA reference database (v138) using the QIIME2 classify-sklearn algorithm to obtain taxonomic classifications and relative abundance profiles [38].

Microbial α-diversity was evaluated using the Chao1, Shannon, Simpson, and Good’s coverage indices based on the ASV abundance table. Differences in microbial community composition (β-diversity) were assessed using weighted UniFrac distance matrices and visualized by principal coordinates analysis (PCoA). Statistical significance of community dissimilarities among treatment groups was evaluated using permutational multivariate analysis of variance (PERMANOVA, 999 permutations). Differentially abundant microbial taxa were identified using linear discriminant analysis effect size (LEfSe, v1.0), whereas differences in the relative abundance of individual taxa between treatment groups were evaluated using Student’s *t*-test where appropriate [39].

### 2.7. Statistical Analysis

All experimental data are presented as the mean ± standard deviation (SD). Data processing and statistical analyses were performed using WPS Office (v12.1.0.21541), Microsoft Excel (v2512, 64-bit), and SPSS Statistics (v26.0.0.0). Prior to analysis, all datasets were tested for normality and homogeneity of variance.

To comprehensively evaluate the effects of exposure concentration, exposure duration, and developmental stage, three-way analysis of variance (three-way ANOVA) was performed with concentration, time, and instar as fixed factors, including all main effects and interaction terms. For normally distributed data, one-way analysis of variance (one-way ANOVA) followed by Tukey’s honestly significant difference (HSD) post hoc test was used to determine significant differences among treatment groups within the same instar and exposure duration. Two-tailed Student‘s *t*-test was applied for selected pairwise comparisons, including comparisons between 7-day and 21-day exposure groups and between different concentration groups. For non-normally distributed data, the Kruskal–Wallis non-parametric test was applied.

Statistical significance was defined as *p* < 0.05. All statistical analyses were conducted using SPSS Statistics (v26.0.0.0).

## 3. Results

### 3.1. Retention and Distribution of PS-MPs in the Gastrointestinal Tract of 5th- and 6th-Instar T. tridentatus

**Figure 1:**
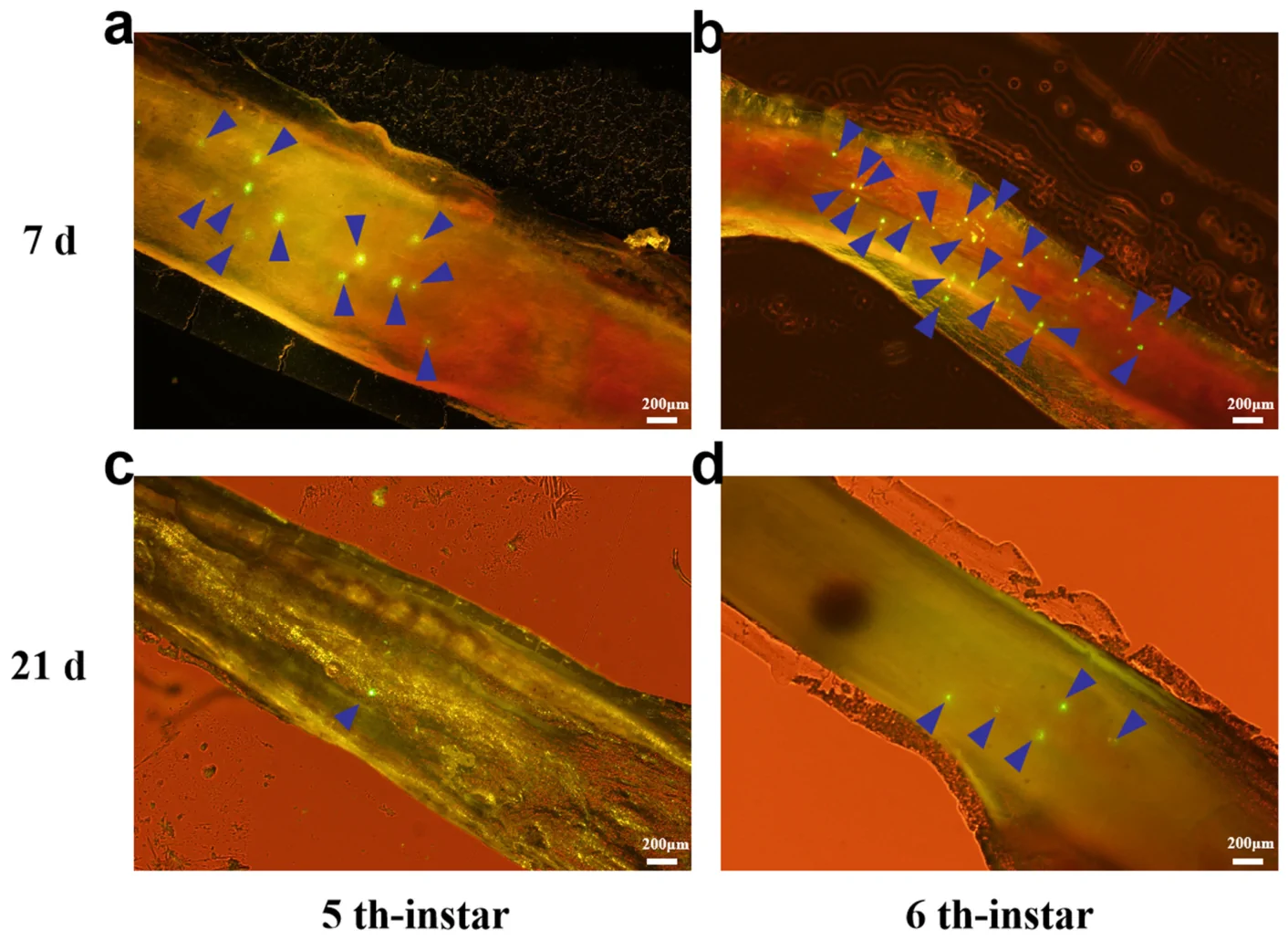
Retention of PS-MPs in the midgut of juvenile *Tachypleus tridentatus* under high-concentration (10^4^ particles/L) exposure. (**a**,**b**) Midgut retention of PS-MPs in 5th- and 6th-instar juveniles after 7 days of exposure, respectively; (**c**,**d**) Midgut retention of PS-MPs in 5th- and 6th-instar juveniles after 21 days of exposure, respectively. The positions indicated by blue triangles represent fluorescent signals. Scale bar = 200 μm.

Fluorescence microscopy suggested the presence and distribution of fluorescent PS-MPs in the gastrointestinal tract of 5th- and 6th-instar juvenile *T. tridentatus* after both 7 and 21 days of exposure (Figure 1). Fluorescent particles were predominantly localized in the midgut, indicating that this region is the principal site of particle retention within the digestive tract (Figure S2).

Qualitatively, fluorescence signals were more intense in intestines containing abundant digesta than in those with little or no intestinal contents, suggesting that gut contents may facilitate the temporary retention of PS-MPs during digestion. In addition, stronger fluorescence signals were generally observed in the high-concentration exposure groups than in the low-concentration groups at the same exposure duration, indicating that particle occurrence in the gastrointestinal tract increased with exposure concentration.

Differences between 5th-instar and 6th-instar juveniles were also observed. Under comparable exposure conditions, 6th-instar juveniles generally exhibited stronger intestinal fluorescence signals than 5th-instar juveniles. As fluorescence microscopy provides qualitative rather than quantitative evidence, these observations should be interpreted as differences in the apparent distribution and retention of PS-MPs rather than as direct measurements of particle burden. Nevertheless, the results suggest that both exposure concentration and developmental stage influence the gastrointestinal occurrence of PS-MPs in juvenile horseshoe crabs.

### 3.2. Effects of PS-MPs on Antioxidant Responses and Innate Immune Parameters in the Gastrointestinal Tract of Juvenile T. tridentatus

**Figure 2:**
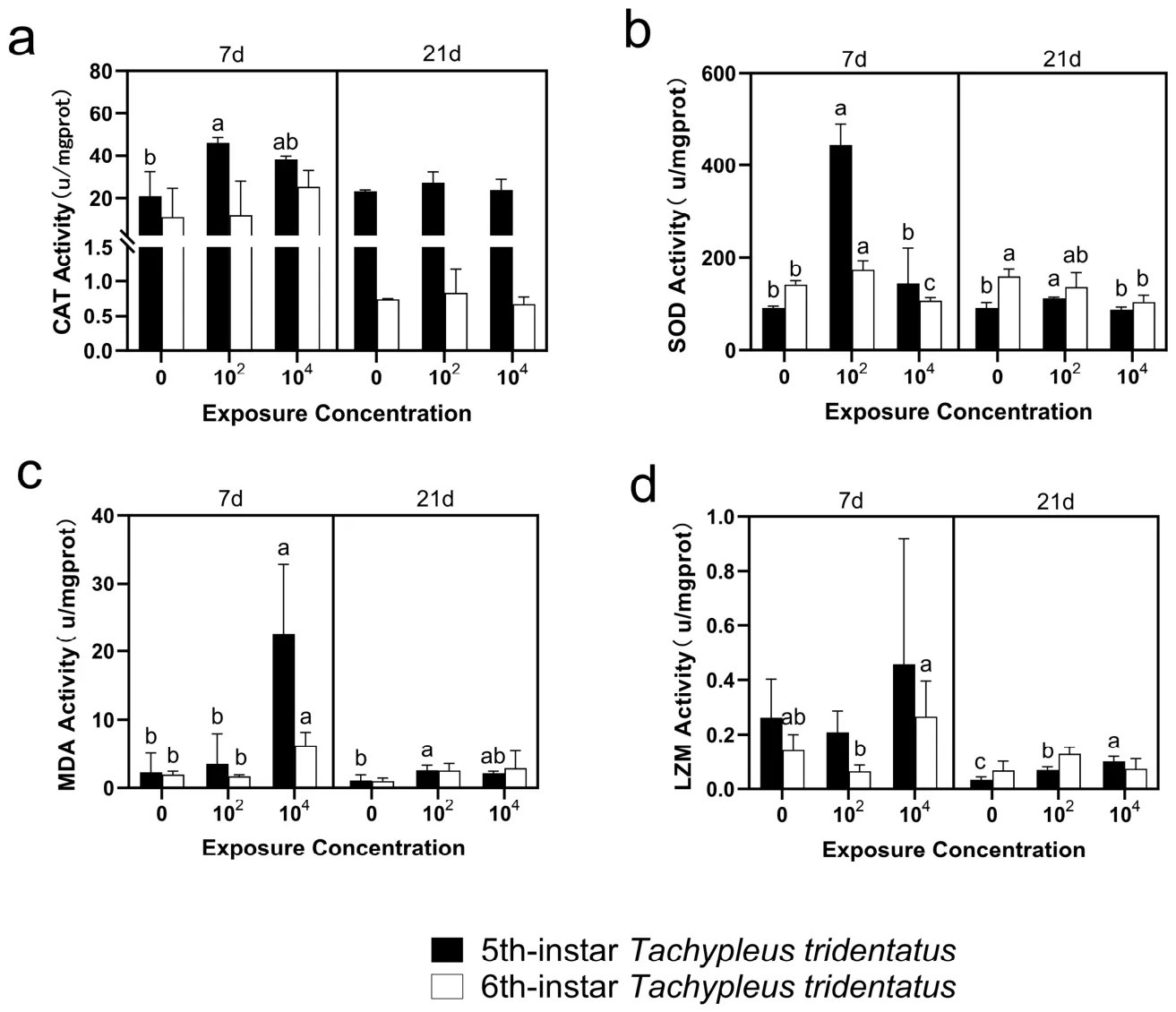
Effects of PS-MP exposure on oxidative stress and immune-related enzyme activities in the gastrointestinal tract of juvenile *Tachypleus tridentatus*. (**a**) Catalase (CAT) activity; (**b**) Superoxide dismutase (SOD) activity; (**c**) Malondialdehyde (MDA) content; (**d**) Lysozyme (LZM) activity. Values are presented as mean ± SD (*n* = 3 biological replicates per group, each pooled from three individuals; each replicate corresponds to an independent culture tank). Different lowercase letters (a, b, c) above the plots indicate significant differences among PS-MP concentrations within the same instar and exposure duration according to one-way ANOVA followed by Tukey’s HSD test (*p* < 0.05). Groups sharing the same letter are not significantly different.

Exposure to PS-MPs significantly affected antioxidant defense (*p* < 0.05), lipid peroxidation (*p* < 0.05), and innate immune responses (*p* < 0.05) in the gastrointestinal tract of juvenile *T. tridentatus*, with responses varying according to exposure concentration, duration, and developmental stage (Figure 2). Three-way ANOVA was performed on SOD, MDA, CAT, and LZM with concentration, time, and instar as fixed factors to evaluate main effects and all two- and three-way interactions (Table S1). For SOD, the main effects of concentration (*p* < 0.001), time (*p* < 0.001), and instar (*p* = 0.009) were all significant, as was the concentration × instar interaction (*p* < 0.001). Similarly, MDA showed significant main effects of concentration (*p* < 0.001), time (*p* < 0.001), and instar (*p* = 0.006), and a significant concentration × instar interaction (*p* = 0.014). For CAT, the main effects of instar (*p* < 0.001) and time (*p* = 0.001) were significant, whereas the concentration × instar interaction was not (*p* = 0.236). For LZM, only the main effect of time reached significance (*p* = 0.005). One-way ANOVA followed by Tukey’s HSD post hoc test was then used to identify specific differences among concentration groups within each instar and exposure duration.

Both CAT and SOD exhibited rapid antioxidant responses during early exposure. After 7 days, CAT activity increased with PS-MP exposure in 5th-instar juveniles, reaching a significant 2.20-fold increase over the control in the low-concentration group, whereas no significant changes were observed in 6th-instar juveniles. SOD activity showed greater sensitivity than CAT. In 5th-instar juveniles, SOD activity increased significantly to 4.86-fold and 1.57-fold of the control under low- and high-concentration exposure, respectively. In contrast, 6th-instar juveniles displayed only a slight increase under low-concentration exposure (1.23-fold of the control), whereas SOD activity declined under high-concentration exposure (0.76-fold of the control). After 21 days, CAT activity no longer differed significantly among treatments, whereas SOD activity remained elevated only in the low-concentration group of 5th-instar juveniles but was significantly suppressed in 6th-instar juveniles exposed to the high concentration (0.66-fold of the control). Notably, basal SOD activity was consistently higher in 6th-instar than in 5th-instar control animals.

Changes in MDA content indicated that oxidative damage was most pronounced during the early exposure period. After 7 days, MDA levels increased markedly in both instars, reaching 9.84-fold of the control in 5th-instar juveniles and 3.23-fold in 6th-instar juveniles under high-concentration exposure. Following 21 days of exposure, MDA levels generally declined, with a significant increase persisting only in the low-concentration group of 5th-instar juveniles (2.42-fold of the control), suggesting a partial physiological acclimation to prolonged PS-MP exposure.

Innate immune responses, as reflected by lysozyme (LZM) activity, were comparatively moderate and exhibited distinct instar-specific patterns. In 5th-instar juveniles, LZM activity showed no significant changes after 7 days but increased significantly after 21 days in both exposure groups, reaching 2.07-fold and 3.02-fold of the control in the low- and high-concentration treatments, respectively. In contrast, 6th-instar juveniles exhibited a transient increase in LZM activity after 7 days (1.85-fold of the control under high-concentration exposure), whereas no significant differences were detected after 21 days. Overall, PS-MP exposure elicited concentration-, exposure duration-, and instar-dependent physiological responses.

### 3.3. PS-MPs Reduced Gut Microbial Diversity

**Figure 3:**
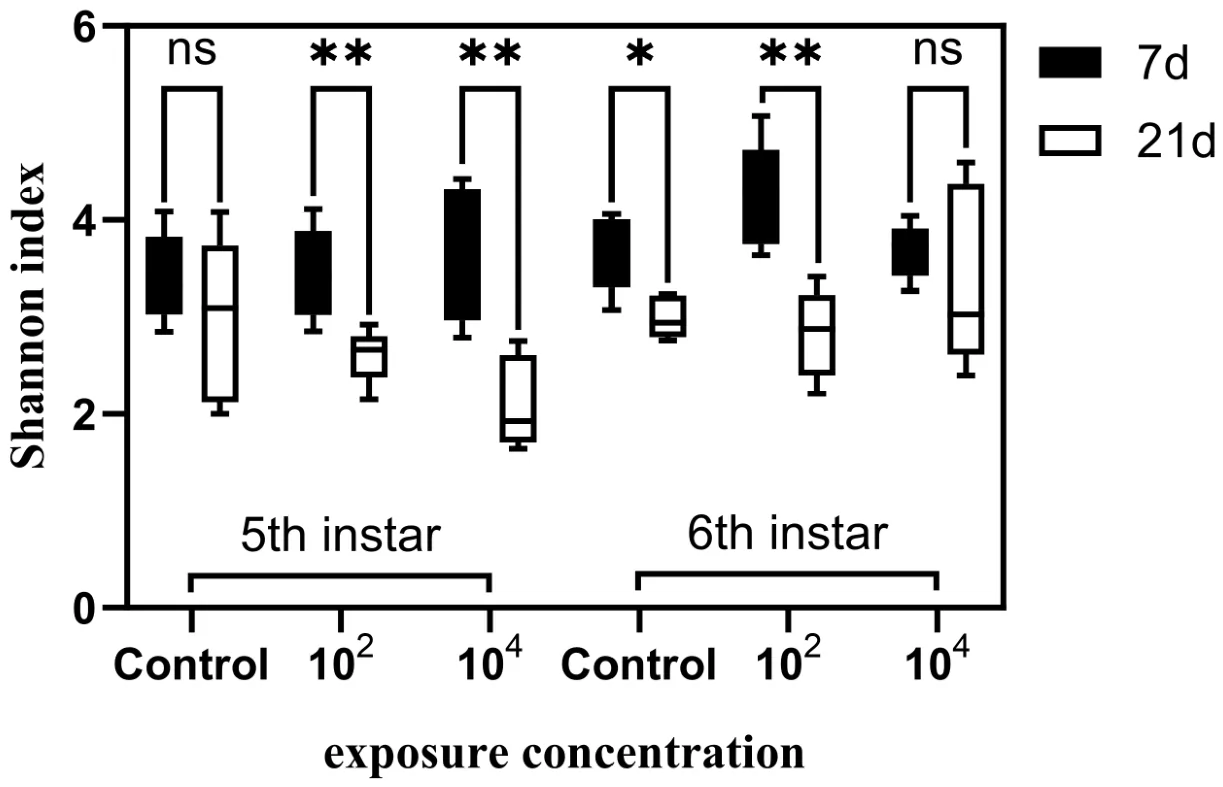
Effects of polystyrene microplastics (PS-MPs; 0, 10^2^, and 10^4^ particles·L^−1^) on the Shannon diversity index of the gut microbiota in juvenile *Tachypleus tridentatus* at two developmental stages (5th and 6th instars) and two exposure durations (7 and 21 days). Black and white boxplots represent the 7-day and 21-day exposure groups, respectively. Values are presented as mean ± SD (*n* = 5 individual biological replicates per group). Statistical significance between the 7-day and 21-day groups at the same concentration and instar was determined by two-tailed *t*-test. ns indicates not significant (*p* > 0.05); * *p* < 0.05; ** *p* < 0.01. Exact *p* values are shown above the brackets.

**Figure 4:**
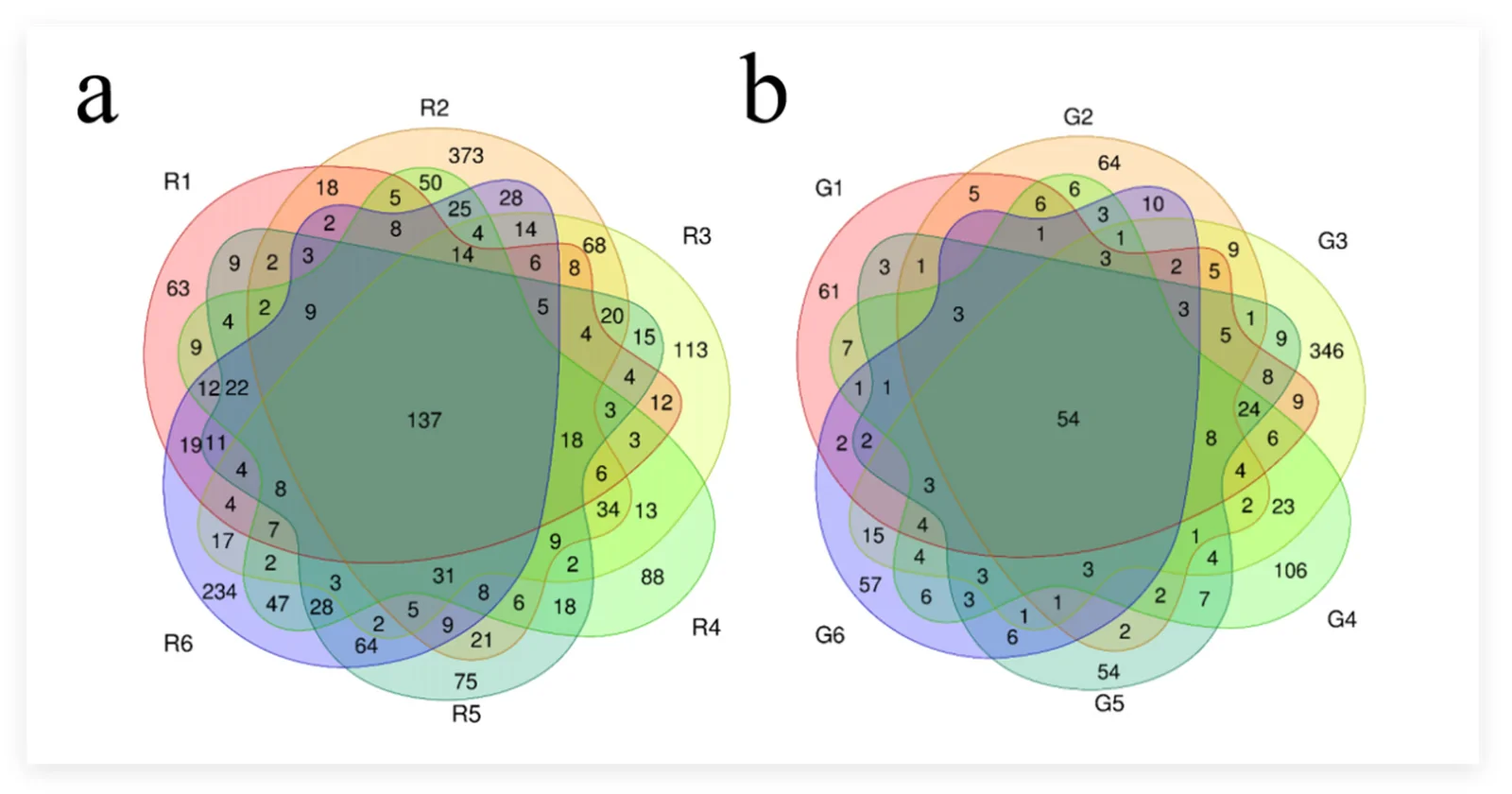
Venn diagrams showing shared and unique ASVs among gastrointestinal microbial communities of juvenile *Tachypleus tridentatus* under different PS-MP exposure durations. (**a**) Shared and unique ASVs among 5th- and 6th-instar juveniles under control, low-concentration, and high-concentration PS-MP exposure after 7 days; (**b**) Shared and unique ASVs among 5th- and 6th-instar juveniles under control, low-concentration, and high-concentration PS-MP exposure after 21 days. Numbers in overlapping regions indicate shared ASVs among groups, whereas numbers in non-overlapping regions represent unique ASVs within each group. Sample group definitions: R-series (7-day exposure groups): R1–R3 represent 6th-instar juveniles (*Tachypleus tridentatus*) (R1: control, R2: low-concentration PS-MPs, R3: high-concentration PS-MPs); R4–R6 represent 5th-instar juveniles (R4: control, R5: low-concentration PS-MPs, R6: high-concentration PS-MPs).

Rarefaction analysis indicated that sequencing depth was sufficient to capture the gut microbial diversity of all samples, as richness and diversity curves approached saturation and Good’s coverage values were close to 1.0 (Figure S3). PS-MP exposure resulted in varied effects on gut microbial richness and diversity in juvenile *T. tridentatus*, depending on exposure duration, concentration, and developmental stage (Table 1, Figure 3). For 5th-instar juveniles, Shannon diversity showed no significant change after 7 days (*p* > 0.05 for all comparisons), but decreased after 21 days in both low- and high-concentration groups (two-tailed *t*-test, *p* < 0.05 and *p* < 0.01, respectively; Figure 3). For 6th-instar juveniles, no significant differences in Shannon diversity were detected between 7-day and 21-day exposure groups at any concentration (*p* > 0.05; Figure 3). Chao1 and Simpson indices followed similar trends, with reductions being more pronounced in 5th-instar juveniles after prolonged exposure (Table 1). It should be noted that certain treatment groups, such as 6th-instar juveniles exposed to low concentration for 7 days (R2), exhibited higher Chao1 and Shannon indices than their respective controls (R1), although the variation among individual replicates was also large. This suggests that short-term low-concentration exposure did not uniformly reduce microbial diversity across all conditions. However, the most consistent and biologically meaningful reductions in diversity indices were observed after 21 days, particularly in 5th-instar juveniles at high concentration (G6), where Chao1 (75.87 ± 9.92), Shannon (2.11 ± 0.48), and Simpson (0.57 ± 0.14) reached the lowest values among all groups. Consistent with the diversity indices, ASV analysis revealed a marked decline in both shared and unique ASVs following prolonged exposure (Figure 4, Figure S4). The number of core ASVs shared among all treatment groups decreased from 137 after 7 days to 54 after 21 days, demonstrating progressive changes in the core gut microbiota. Collectively, these results indicate that chronic PS-MP exposure mainly reduced gut microbial diversity and altered community stability in juvenile *T. tridentatus*.

### 3.4. Differential Bacterial Taxa Identified by LEfSe

**Figure 5:**
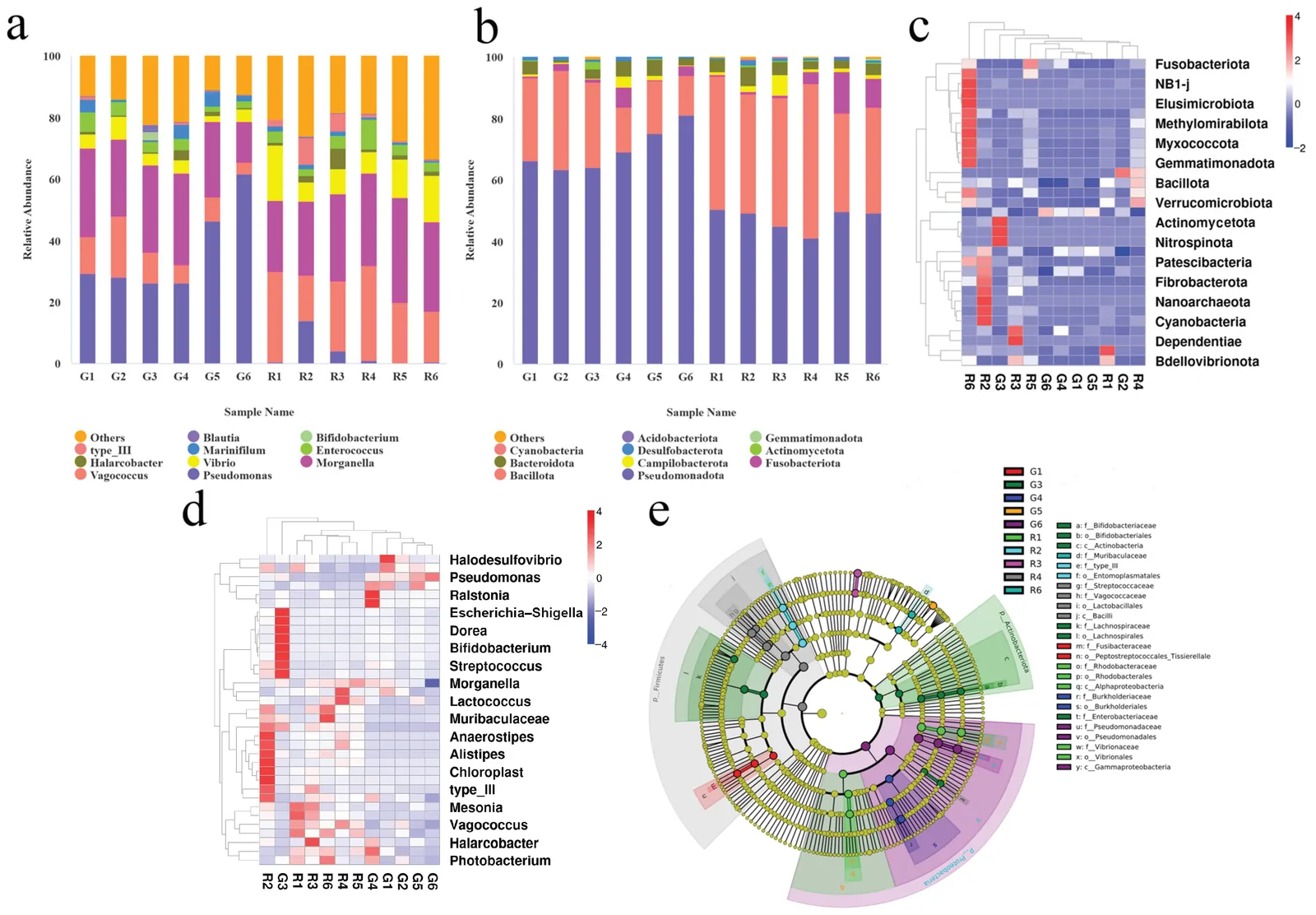
Relative abundance, clustering heatmaps, and LEfSe biomarker analysis of the gut microbiota in juvenile *Tachypleus tridentatus*. (**a**) Stacked bar plot of relative abundance at the phylum level; (**b**) Stacked bar plot of relative abundance at the genus level; (**c**) Hierarchical clustering heatmap at the phylum level (top 35 most abundant bacterial phyla); (**d**) Hierarchical clustering heatmap at the genus level (top 35 most abundant bacterial genera); (**e**) LEfSe cladogram showing taxonomic biomarkers of gut microbial communities. In the heatmaps, red indicates higher relative abundance, and blue indicates lower relative abundance. In the cladogram, taxa are shown from the inner to outer rings, representing the phylum, class, order, family, and genus levels. Yellow nodes indicate taxa with no significant differences among groups, while nodes in other colors represent significantly different taxa among treatment groups. Sample group definitions: R-series (7-day exposure groups): R1–R3 represent 6th-instar juveniles (*Tachypleus tridentatus*) (R1: control, R2: low-concentration PS-MPs, R3: high-concentration PS-MPs); R4–R6 represent 5th-instar juveniles (R4: control, R5: low-concentration PS-MPs, R6: high-concentration PS-MPs).

Gut microbial composition was dominated by Pseudomonadota, Bacillota, and Bacteroidota across all treatments (Figure 5a,b and Figure S5). PS-MP exposure induced marked shifts in the relative abundance of these dominant phyla, characterized by enrichment of Pseudomonadota and depletion of Bacillota, particularly after 21 days of high-concentration exposure. These changes were more pronounced in 5th-instar juveniles, in which Pseudomonadota increased to 81.13% while Bacillota decreased to 12.85%. At the genus level, *Pseudomonas*, *Morganella*, and *Vagococcus* predominated the intestinal microbiota (Figure S5). Prolonged PS-MP exposure resulted in a pronounced enrichment of *Pseudomonas* accompanied by a progressive decline in *Vagococcus*, with the strongest responses observed in 5th-instar juveniles. Heatmap analysis further indicated widespread taxonomic restructuring following PS-MP exposure, with Bacillota-associated genera showing the greatest reductions, whereas multiple taxa belonging to Pseudomonadota became enriched across treatment groups (Figure 5c,d and Figure S6).

### 3.5. LEfSe Analysis of Key Discriminatory Taxa

LEfSe analysis identified distinct microbial biomarkers associated with PS-MP exposure across developmental stages and exposure durations (Figure 5e and Figure S7). Bacillota, Pseudomonadota, and Actinomycetota were the principal discriminatory phyla, whereas Bacilli, Alpha Pseudomonadota, and Gamma Pseudomonadota were the dominant discriminatory classes. At lower taxonomic levels, control groups were characterized by several taxa associated with normal gut microbiota, including *Lactobacillales*, *Vagococcus*, and *Lactococcus*, whereas prolonged high-concentration exposure enriched taxa such as *Pseudomonas*, Pseudomonadaceae, Enterobacteriaceae, *Blautia*, and *Bifidobacterium*. *t*-test analysis suggested significant alterations in the abundance of key bacterial genera, particularly the concentration-dependent enrichment of *Pseudomonas* after 21 days of exposure in 5th-instar juveniles (*p* = 0.034), together with significant changes in *Blautia*, *Escherichia–Shigella*, *Bacteroides*, and *Cohaesibacter* (Figure S8).

### 3.6. Overall Shifts in Microbial Community Structure

Principal coordinate analysis based on weighted UniFrac distances indicated clear separation of gut microbial communities according to PS-MP concentration, exposure duration, and developmental stage (Figure S9). PC1 and PC2 explained 66.28% and 15.07% of the total variation, respectively, accounting for 81.35% of the cumulative variance. Samples from the 7- and 21-day exposure groups formed distinct clusters, with the greatest separation observed among 5th-instar juveniles after 21 days. These results indicate that both prolonged exposure and developmental stage contributed substantially to gut microbial community divergence following PS-MP exposure.

## 4. Discussion

The present study demonstrates the retention characteristics of the emerging marine contaminant PS-MPs in the gastrointestinal tract of juvenile *T. tridentatus* during critical developmental stages (5th and 6th instars) and reveals their combined toxic effects, including oxidative injury, immune suppression, and gut microbial dysbiosis. These findings highlight the complex impacts of PS-MPs on intestinal microecological homeostasis in juvenile *T. tridentatus* and further confirm that exposure concentration, exposure duration, and host developmental stage are key determinants of toxicological responses.

### 4.1. Ingestion, Retention, and Instar-Dependent Retention Characteristics of PS-MPs in the Midgut

In the present study, juvenile *T. tridentatus* primarily ingested PS-MPs via feeding and water intake, with retention observed in the midgut. This uptake pathway is consistent with trophic transfer patterns reported in other aquatic organisms, such as zebrafish and tuna [16,40].

Sultan et al. [16] indicated that nanoplastics can be efficiently transferred through a trophic chain (*Artemia* → zebrafish), resulting in significant gastrointestinal occurrence and a clear bioaccumulation gradient in zebrafish. In the present study, fluorescence microscopy revealed that intestinal tracts containing food residues exhibited higher fluorescence intensity, whereas intestines with little or no food content showed markedly weaker signals. Moreover, strong fluorescent signals were also detected in fecal material, indicating that MPs associated with food particles can be rapidly excreted, although a fraction may remain retained in the gut. As fluorescence microscopy provides qualitative rather than quantitative evidence, the observed fluorescence signals indicate the presence of particles within the intestinal lumen rather than providing a direct measure of particle burden. An observation in the present study was the reduction in fluorescence intensity from 7 to 21 days of exposure. This trend does not necessarily indicate that ingestion decreased over time, but may reflect a dynamic balance between continuous particle ingestion and progressive elimination through normal digestive and excretory processes in juvenile *T. tridentatus*. Under chronic exposure conditions, the intestinal clearance rate could gradually exceed the ingestion rate, leading to a reduction in particle occurrence in the gut over time. Similar excretion kinetics have been reported in other benthic invertebrates, where microplastics are transiently retained in the gut and subsequently excreted [41]. Wang et al. [40] further confirmed that yellowfin tuna (*Thunnus albacares*) from coastal waters of southern Kaohsiung, Taiwan, can directly ingest microplastics through both respiration (filtration and retention via gills) and drinking (uptake of MPs suspended in water), as evidenced by the abundance and morphology of MPs detected in gill and stomach tissues.

Our observations indicate that PS-MP retention exhibits a clear concentration–response relationship, with exposure concentration being a key determinant of intestinal burden. Higher environmental concentrations led to greater ingestion and retention of PS-MPs in the gut. This finding is consistent with Danting Wang et al. [42], who reported that fluorescence intensity of red fluorescent polystyrene nanoplastics in zebrafish larvae increased with rising exposure concentrations. Furthermore, under identical exposure conditions, sixth-instar juveniles exhibited higher retention levels than fifth-instar individuals. This may be attributed to higher feeding rates and greater intestinal content volume in 6th-instar juveniles, which increases the likelihood of particle retention and adhesion within the digestive tract.

The presence of MPs in intestinal contents may further prolong retention time through mucus encapsulation and particle aggregation, forming so-called “intestinal mucus–microplastic aggregates,” thereby exacerbating retention. A similar mechanism was proposed by Hoang and Felix-Kim [41], who observed in juvenile soft-mouth minnows that ingested MPs could be re-ingested during excretion, and that expelled MPs coated with intestinal mucus tended to aggregate. This cyclical ingestion–excretion process, combined with the strong adhesive properties of MPs, ultimately contributes to prolonged retention in the gut. Therefore, the strong fluorescence signals observed in this study are likely attributable to mucus-mediated aggregation and retention of PS-MPs within the intestinal environment.

### 4.2. Instar-Specific Oxidative Imbalance and Intestinal Immune Impairment

Oxidative stress is considered one of the primary mechanisms underlying microplastic toxicity in aquatic organisms. In the present study, PS-MP exposure induced pronounced alterations in the antioxidant defense system of juvenile *T. tridentatus*, suggesting a possible impairment of intestinal redox homeostasis under environmentally relevant exposure conditions. Although reactive oxygen species (ROS) were not directly measured in this study, the transient elevation of CAT and SOD activities during early exposure may be consistent with a compensatory activation of antioxidant defenses in response to elevated ROS levels, whereas the diminished responses after prolonged exposure might suggest that sustained oxidative stress could gradually overwhelm cellular antioxidant capacity [43,44].

The concurrent increase in MDA further demonstrates that antioxidant activation was insufficient to completely prevent membrane lipid peroxidation, particularly in 5th-instar juveniles exposed to higher PS-MP concentrations. As MDA is a reliable indicator of oxidative injury [45], these findings suggest that earlier developmental stages experienced greater oxidative injury, whereas 6th-instar juveniles maintained relatively stronger redox homeostasis. Similar developmental-stage differences were also reflected in innate immune responses. Lysozyme activity showed comparatively moderate changes, yet older juveniles exhibited greater capacity to maintain immune stability under prolonged exposure, suggesting a more effective physiological compensation [46].

Together, these findings demonstrate that the susceptibility of juvenile *T. tridentatus* to PS-MP-induced oxidative stress is strongly influenced by developmental stage. The greater physiological sensitivity of 5th-instar juveniles suggests that early post-hatching development may represent a particularly vulnerable period during which oxidative and immune disturbances could have disproportionate consequences for subsequent growth and survival. The significant concentration × instar interactions for SOD (*p* < 0.001) and MDA (*p* < 0.05) further indicated that 5th-instar juveniles were more susceptible to PS-MP-induced oxidative stress than 6th-instar individuals. Specifically, the SOD response—a key indicator of antioxidant defense activation—was markedly stronger in 5th-instar juveniles upon microplastic exposure, suggesting a more vigorous compensatory effort to counteract reactive oxygen species. Concurrently, the more pronounced elevation of MDA, a stable end-product of lipid peroxidation, implied that 5th-instar juveniles suffered greater oxidative membrane damage despite their heightened antioxidant mobilization. This combined pattern—stronger enzymatic defense but also greater cellular injury—supports the view that the earlier developmental stage is physiologically more vulnerable to oxidative challenge imposed by PS-MPs.

An important observation in the present study is that PS-MP retention did not correspond directly to toxicity severity. Although 6th-instar juveniles consistently exhibited higher intestinal retention of PS-MPs than 5th-instar individuals, the latter showed stronger oxidative stress responses, greater lipid peroxidation, and more pronounced microbial dysbiosis. This discrepancy indicates that body burden alone is insufficient to predict toxicological outcomes in developing *T. tridentatus*.

Several factors may explain this pattern. First, sixth-instar juveniles appeared to possess a more mature antioxidant defense system, as evidenced by their higher basal SOD activity and relatively stable physiological responses, enabling more efficient maintenance of redox homeostasis under chronic PS-MP exposure. Similar developmental-stage differences in antioxidant capacity have been reported in juvenile *T. tridentatus* exposed to plastic-related pollutants [33]. Second, older juveniles likely have a more stable and resilient gut microbial ecosystem, which may buffer the disruptive effects of microplastics on intestinal homeostasis. Third, the stronger fluorescence observed in 6th-instar individuals may partly reflect larger food intake and greater gut content volume rather than a proportionally higher degree of particle internalization into intestinal tissues. Consequently, a substantial fraction of ingested particles may remain within the gut lumen and be eliminated through normal digestive processes, reducing direct contact with epithelial cells.

Collectively, these findings indicate that physiological maturity, rather than microplastic retention per se, is the primary determinant of toxic susceptibility during juvenile development of *T. tridentatus*. This interpretation is consistent with recent evidence showing that developmental stage strongly influences the biological responses of juvenile *T. tridentatus* to polystyrene microplastics [4] and supports the broader view that host physiological condition is a key modifier of microplastic toxicity in marine invertebrates [3].

### 4.3. PS-MPs Impair the Gut Microbiota Through Oxidative Stress-Mediated Alterations of the Intestinal Microenvironment

PS-MP exposure markedly altered the diversity and composition of the intestinal microbiota in juvenile *T. tridentatus*, with 5th-instar juveniles exhibiting greater reductions in microbial richness and diversity than 6th-instar individuals. The decline in Chao1 and Shannon indices, together with the reduction in shared ASVs, indicates that prolonged PS-MP exposure progressively destabilized the intestinal microbial community. Rather than representing isolated microbial changes, these alterations likely reflect disturbances in the intestinal microenvironment initiated by oxidative stress.

Increasing evidence suggests that excessive reactive oxygen species (ROS) generated during microplastic exposure act not only as cytotoxic molecules but also as ecological drivers shaping gut microbial communities. Elevated ROS levels can alter intestinal oxygen availability, mucus composition, epithelial permeability, and nutrient gradients, thereby creating selective conditions that favor facultative aerobic or opportunistic bacteria while suppressing beneficial anaerobic commensals. In the present study, the marked enrichment of Pseudomonadota, accompanied by the decline of Bacillota, is consistent with microbial signatures of intestinal dysbiosis reported in aquatic organisms exposed to environmental contaminants [47]. Similar community shifts have frequently been associated with impaired intestinal barrier function and increased susceptibility to inflammation.

At the genus level, the expansion of *Pseudomonas* and the simultaneous reduction of beneficial genera such as *Lactococcus* further support this ecological transition. Beneficial lactic acid bacteria contribute to pathogen exclusion, maintenance of epithelial integrity, and immune homeostasis, whereas opportunistic genera including *Pseudomonas* readily proliferate under oxidative and inflammatory conditions. Therefore, the microbial alterations observed here are unlikely to represent direct toxic effects of PS-MPs on bacteria alone but instead appear to arise from reactive oxygen species (ROS)-mediated remodeling of the intestinal microenvironment, linking oxidative stress with microbial dysbiosis.

Collectively, these findings suggest that oxidative stress represents an upstream driver connecting microplastic exposure with gut microbial instability in juvenile *T. tridentatus*.

### 4.4. Gut Dysbiosis Amplifies Oxidative Imbalance Through Impairment of the Gut–Microbiota–Immune Axis

The present study further suggests that intestinal dysbiosis is not merely a consequence of PS-MP exposure but may actively amplify host toxicity through disruption of the gut-microbiota-immune axis. Following oxidative stress-induced alterations of the intestinal environment, the loss of beneficial bacteria together with the enrichment of opportunistic pathogens is expected to impair mucosal immune function and intestinal barrier integrity, thereby establishing a self-reinforcing cycle of intestinal injury.

This hypothesis is supported by the LEfSe analysis, which revealed the emergence of distinct biomarkers associated with potentially pathogenic taxa after prolonged high-concentration exposure. For example, Enterobacteriaceae, detected as a characteristic biomarker in 6th-instar juveniles after 21 days of high-concentration exposure, is widely recognized as an indicator of intestinal dysbiosis and inflammatory disorders. Excessive proliferation of Enterobacteriaceae can activate host inflammatory pathways, aggravate epithelial damage, and further disturb microbial homeostasis [48]. Likewise, the increased abundance of Campilobacterota, which contains numerous gastrointestinal pathogens [49], suggests that chronic PS-MP exposure may increase susceptibility to intestinal infection.

Conversely, beneficial taxa including *Lactococcus*, which produce antimicrobial metabolites and contribute to colonization resistance [50], declined following PS-MP exposure. The reduction of these protective microorganisms may weaken pathogen exclusion and compromise innate immune defenses, consistent with the altered lysozyme responses observed in the present study.

Taken together, our findings support a positive feedback mechanism linking oxidative stress, microbial dysbiosis, and immune dysfunction. Initial PS-MP retention triggers excessive ROS production, which impairs intestinal epithelial homeostasis and alters the gut microenvironment. These changes selectively promote opportunistic bacteria while suppressing beneficial commensals. The resulting dysbiosis may further impair immune function and intestinal barrier integrity, thereby enhancing oxidative injury and sustaining chronic intestinal inflammation. This oxidative stress–microbiota–immune feedback loop provides a mechanistic framework for understanding the chronic gastrointestinal toxicity of PS-MPs in juvenile *T. tridentatus*.

### 4.5. Proposed Mechanism Underlying PS-MP-Induced Intestinal Alterations in Juvenile T. tridentatus

Based on the present physiological and microbial findings, a conceptual mechanism model was proposed to illustrate the potential responses of juvenile *T. tridentatus* to PS-MP exposure. Following ingestion, PS-MPs may be retained in the midgut and interact with intestinal tissues, leading to oxidative imbalance characterized by reduced antioxidant enzyme activities and increased lipid peroxidation. Meanwhile, alterations in lysozyme activity suggest that PS-MPs may influence innate immune responses. In parallel, PS-MP exposure reshaped gut microbial communities, including decreased relative abundances of beneficial taxa and enrichment of potential opportunistic bacteria. These interconnected changes may collectively contribute to impaired intestinal homeostasis.

However, this model represents a hypothesis based on observed associations rather than confirmed causal pathways. Further investigations involving direct measurements of ROS generation, intestinal barrier-related genes, inflammatory signaling pathways, and microbial functional analyses are required to validate the proposed mechanisms. A limitation is that the nominal particle size reported by the manufacturer may differ from the effective size in the exposure medium, as particle size distribution and aggregation state were not quantitatively characterized.

## 5. Conclusions

This study demonstrates that environmentally relevant concentrations of PS-MPs are predominantly retained within the midgut of endangered juvenile *Tachypleus tridentatus* and impair intestinal homeostasis via three interconnected pathways: oxidative stress, innate immune dysfunction, and gut microbial dysbiosis. These toxic responses exhibit clear concentration-, exposure duration-, and instar-dependent patterns, with 5th-instar juveniles showing consistently higher susceptibility than 6th-instar individuals, suggesting that early ontogeny represents a critical vulnerability window for this ancient marine arthropod. By integrating physiological and microbial endpoints, this work establishes a mechanistic framework for instar-specific microplastic toxicity in benthic arthropods; our findings provide empirical support for refining ecological risk assessments of coastal microplastic pollution and inform targeted conservation strategies for *T. tridentatus* in microplastic-impacted intertidal ecosystems, with broader implications for coastal benthic biodiversity protection under anthropogenic disturbance. However, the present single-pollutant laboratory results may not fully represent the responses under environmentally realistic co-exposure conditions. Future research incorporating multi-omics approaches, long-term exposure regimes, and field-relevant co-exposure scenarios will help translate laboratory toxicological findings into population- and community-level ecological outcomes, further strengthening the scientific basis for marine invertebrate conservation.

## Figures and Tables

**Table 1:** Effects of PS-MPs on the diversity indices of gut bacterial communities in *Tachypleus tridentatus*. Definition of sample group codes used in this study. R-series (7-day exposure groups): R1–R3 represent 6th-instar juveniles of *Tachypleus tridentatus* (R1: control; R2: low-concentration PS-MPs exposure; R3: high-concentration PS-MP exposure); R4–R6 represent 5th-instar juveniles (R4: control; R5: low-concentration PS-MP exposure; R6: high-concentration PS-MP exposure). G-series (21-day exposure groups): G1–G3 represent 6th-instar juveniles (G1: control; G2: low-concentration PS-MP exposure; G3: high-concentration PS-MP exposure); G4–G6 represent 5th-instar juveniles (G4: control; G5: low-concentration PS-MP exposure; G6: high-concentration PS-MP exposure).

	Chao1	Shannon	Simpson
R1	185.02 ± 27.09	3.66 ± 0.34	0.84 ± 0.04
R2	354.62 ± 102.87	4.25 ± 0.50	0.85 ± 0.04
R3	226.62 ± 39.51	3.68 ± 0.25	0.83 ± 0.03
R4	235.20 ± 30.39	3.41 ± 0.41	0.78 ± 0.07
R5	229.01 ± 41.17	3.45 ± 0.42	0.80 ± 0.08
R6	272.37 ± 112.87	3.76 ± 0.66	0.82 ± 0.08
G1	88.89 ± 11.99	3.00 ± 0.22	0.76 ± 0.05
G2	87.64 ± 10.85	2.83 ± 0.46	0.73 ± 0.09
G3	166.31 ± 103.65	3.41 ± 0.93	0.75 ± 0.12
G4	120.23 ± 29.01	2.96 ± 0.85	0.72 ± 0.17
G5	95.35 ± 17.09	2.61 ± 0.28	0.70 ± 0.05
G6	75.87 ± 9.92	2.11 ± 0.48	0.57 ± 0.14

## Data Availability

The raw data supporting the conclusions of this article will be made available by the authors on request.

## Supplementary Materials

The following supporting information can be downloaded at https://www.mdpi.com/article/10.3390/toxics14090797/s1. Figure S1: Experimental materials and rearing system; Figure S2: Fluorescence micrographs of different tissues from 5th- and 6th-instar larvae; Figure S3: Rarefaction curves; Figure S4: Petal diagram (Venn diagram) of gut microbiota in *Tachypleus tridentatus*; Figure S5: Cumulative histogram of relative abundance; Figure S6: Abundance heatmaps of the top 35 bacterial phyla or genera; Figure S7: LDA score distribution histogram of biomarkers for the intestinal microbiota; Figure S8: *T*-test analysis diagram (pertaining to classification); Figure S9: Principal component analysis (PCA)of gut microbiota in *Tachypleus tridentatus*; Table S1: Summary of three-way ANOVA results for physiological parameters in juvenile *Tachypleus tridentatus* exposed to PS-MPs.
