# Supplementary material for: Polystyrene Microplastics Impair Intestinal Homeostasis in Juvenile Tachypleus tridentatus Through Oxidative Imbalance and Gut Microbiota Dysregulation
Source: Toxics. 2026 Sep 9;14(9):797. doi: 10.3390/toxics14090797 (PMC13611210; doi:10.3390/toxics14090797)
Supplement: Supplementary file 1 [file toxics-14-00797-s001.zip › toxics-4500879-supplementary.pdf]

Supplementary material for

Polystyrene Microplastics Impair Intestinal Homeostasis in Juvenile *Tachypleus tridentatus* through Oxidative Imbalance and Gut Microbiota Dysregulation

Figure and Table Supplementary Files of the Manuscript

Figure S1 Experimental materials and rearing system. (A) 5th-instar juvenile *Tachypleus tridentatus* used in the experiment; (B) 6th-instar juvenile *Tachypleus tridentatus* used in the experiment (yellow scale bars indicate body size); (C) laboratory aquaculture system during the exposure experiment.

Figure S2 Fluorescence micrographs of different tissues from 5th- and 6th-instar larvae.

(a) Midgut of 5th-instar larva. (b) Carapace of 5th-instar larva. (c) Gill of 5th-instar larva. (d) Hindgut of 5th-instar larva. (e) Midgut of 6th-instar larva. (f) Hindgut of 6th-instar larva. (g) Carapace of 6th-instar larva. (h) Foregut of 6th-instar larva.(i-j) Fluorescent signals detected in fecal samples.

Figure S3 Rarefaction curves. (a) Chao1 index; (b) Shannon index; (c) Simpson index; (d) Good's coverage.

Figure S4 Petal diagram (Venn diagram) of gut microbiota in *Tachypleus tridentatus*. Each circle represents a sample group; numbers in overlapping areas indicate shared ASVs between groups, and numbers in non-overlapping areas denote unique ASVs per group. Each petal represents one sample group, with different colors distinguishing groups.The central number is the total ASVs shared across all samples,and the numbers on the petals are group-specific unique ASVs.The diagram summarizes ASV counts across groups to illustrate shared or unique bacterial species characteristics.Sub-figures(a)-(d)and(e)-(h)correspond to different time points,instar stages,and concentration combinations:(a)7 d, 5th-instar: control, low-concentration, high-concentration; (b)7 d, 6th-instar: control, low-concentration, high-concentration; (c)21 d, 5th-instar: control, low-concentration, high-concentration; (d)21 d, 6th-instar: control, low-concentration, high-concentration; (e)7 d, 5th- and 6th-instar: control, low-concentration, high-concentration; (f)21 d, 5th- and 6th-instar: control,

low-concentration, high-concentration; (g)7 d and 21 d, 5th-instar: control, low-concentration, high-concentration; (h)7 d and 21 d, 6th-instar: control, low-concentration, high-concentration.

Figure S5 Cumulative histogram of relative abundance. (a) At the phylum level; (b) At the genus level.

Figure S6 Abundance heatmaps of the top 35 bacterial phyla or genera. Red indicates high abundance, blue indicates low abundance. (a) Phylum-level heatmap; (b) genus-level heatmap.

Figure S7 LDA score distribution histogram of biomarkers for the intestinal microbiota. Species with significant differences have LDA scores greater than the set threshold (default threshold: 4.0); the length of the histogram represents the LDA score, indicating the degree of influence of species with significant differences between groups.

Figure S8 *T*-test analysis diagram (pertaining to classification). Displaying *T*-test results for taxa with significant differences between groups.

Figure S9 Principal component analysis (PCA)of gut microbiota in *Tachypleus tridentatus.* The x-and y-axes represent the first two principal components, with percentages indicating the contribution of each component to sample variance. Each dot represents one sample; samples within the same group are colored identically. Closer proximity indicates greater similarity in species composition, while wider separation denotes significant community differences. Sub-figures (a)–(h) correspond to the following group comparisons: (a)7 d, 6th-instar: control, low-concentration, high-concentration; (b) 7 d, 5th-instar: control, low-concentration, high-concentration; (c) 21 d, 6th-instar: control, low-concentration, high-concentration; (d) 21 d, 5th-instar: control, low-concentration, high-concentration; (e) 7 d, 5th- and 6th-instar:

control, low-concentration, high-concentration; (f) 21 d, 5th- and 6th-instar: control, low-concentration, high-concentration; (g) 7 d and 21 d, 6th-instar: control, low-concentration, high-concentration; (h) 7 d and 21 d, 5th-instar: control, low-concentration, high-concentration.

Table S1 Summary of three-way ANOVA results for physiological parameters in juvenile Tachypleus tridentatus exposed to PS-MPs. Three-way ANOVA was performed with concentration (0, 10^2, 10^4 particles/L), time (7 d, 21 d), and instar (5th, 6th) as fixed factors, including all main effects and interaction terms. Values are presented as F (degrees of freedom) and p-values. Significant p-values (< 0.05) are indicated in bold. CAT: catalase; SOD: superoxide dismutase; MDA: malondialdehyde; LZM: lysozyme.

Figure S1

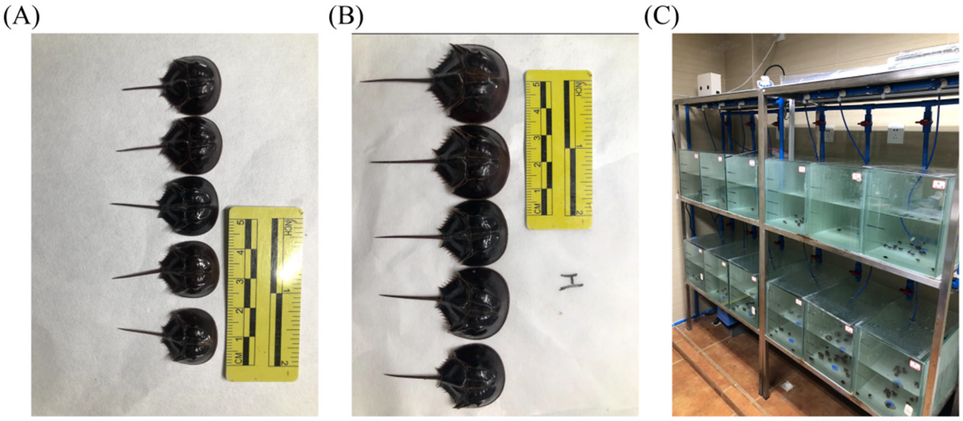

Figure S1 Experimental materials and rearing system.(A) 5th-instar juvenile *Tachypleus tridentatus* used in the experiment; (B) 6th-instar juvenile *Tachypleus tridentatus* used in the experiment (yellow scale bars indicate body size); (C) laboratory aquaculture system during the exposure experiment.

Figure S2

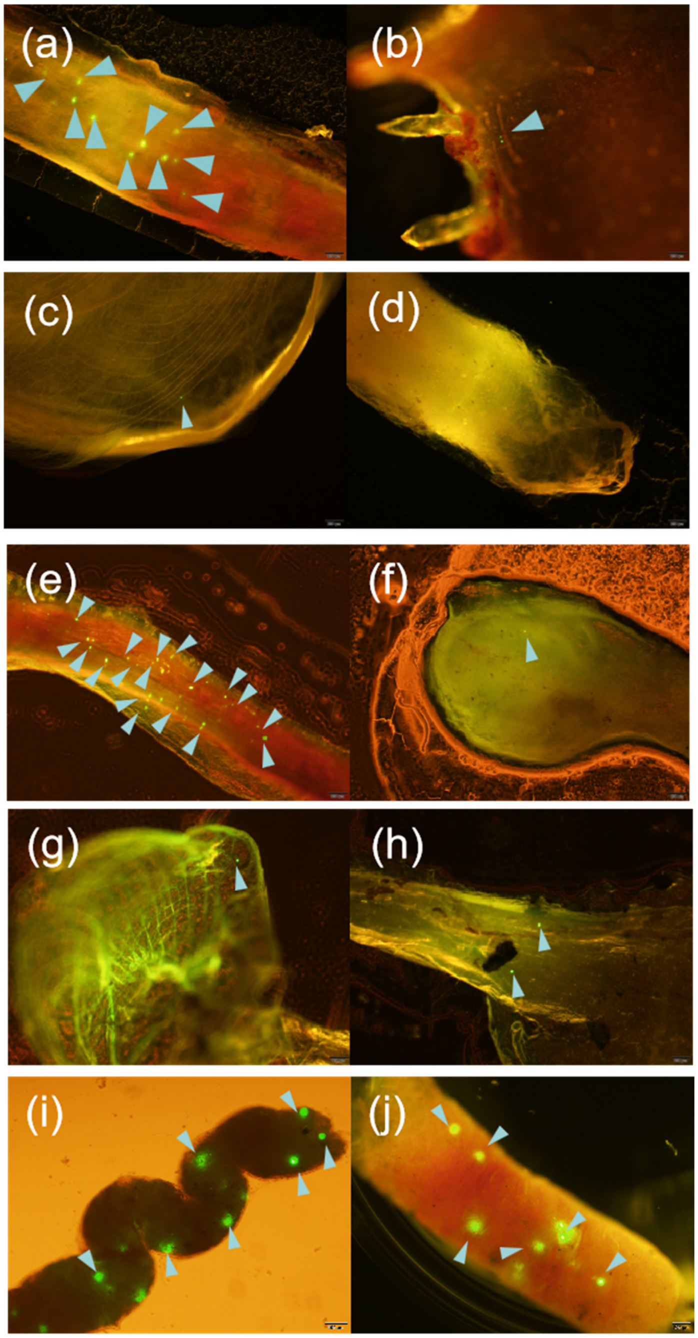

Figure S2 Fluorescence micrographs of different tissues from 5th- and 6th-instar larvae.

(a) Midgut of 5th-instar larva. (b) Carapace of 5th-instar larva. (c) Gill of 5th-instar larva. (d) Hindgut of 5th-instar larva. (e) Midgut of 6th-instar larva. (f) Hindgut of 6th-instar larva. (g) Carapace of 6th-instar larva. (h) Foregut of 6th-instar larva. (i-j) Fluorescent signals detected in fecal samples.

Figure S3

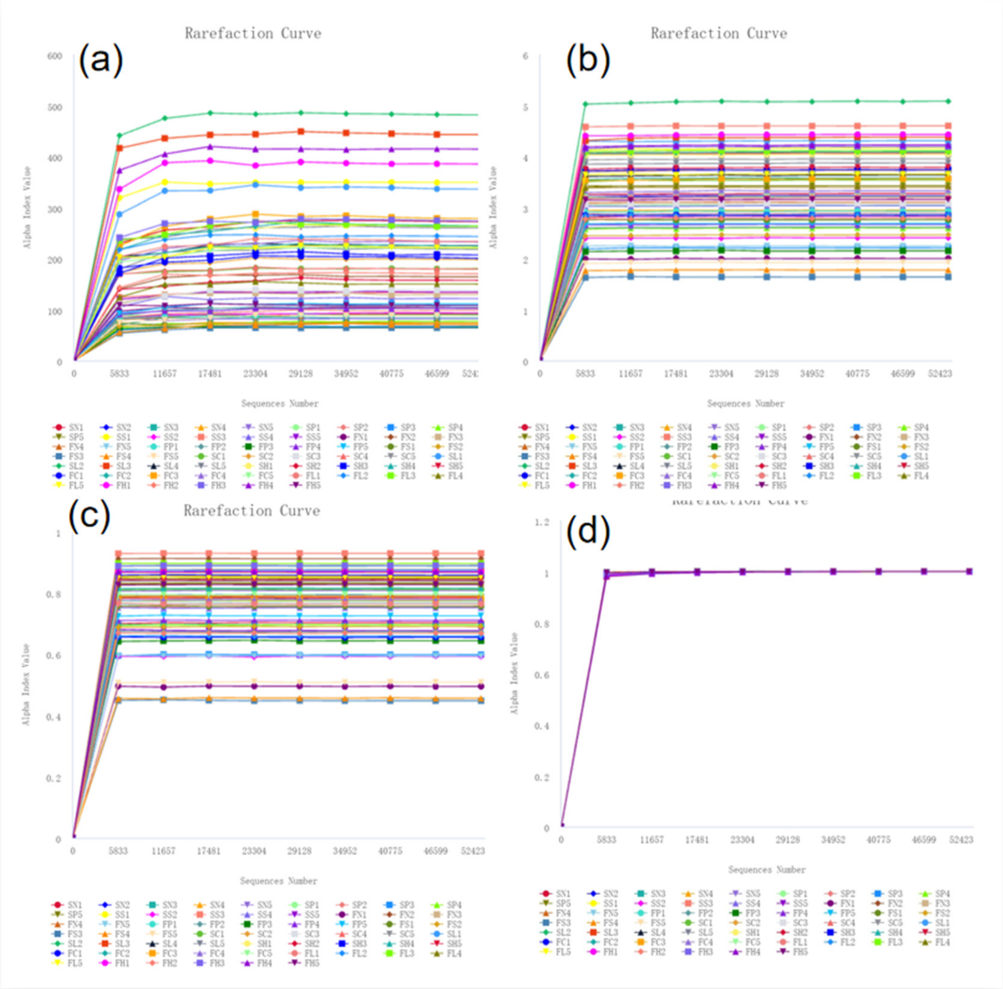

Figure S3 Rarefaction curves. (a) Chao1 index; (b) Shannon index; (c) Simpson index; (d) Good's coverage.

Figure S4

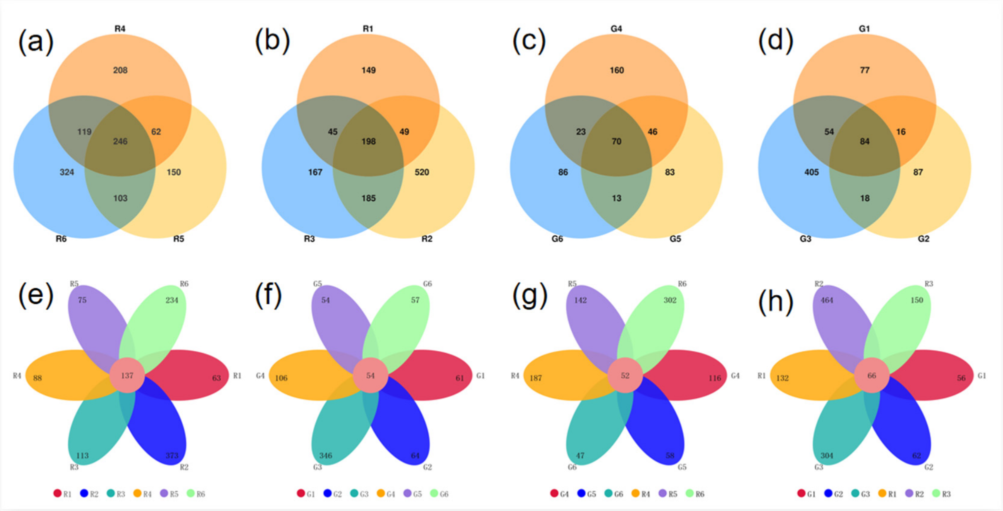

Figure S4 Petal diagram (Venn diagram) of gut microbiota in *Tachypleus tridentatus*. Each circle represents a sample group; numbers in overlapping areas indicate shared ASVs between groups, and numbers in non-overlapping areas denote unique ASVs per group. Each petal represents one sample group, with different colors distinguishing groups.The central number is the total ASVs shared across all samples,and the numbers on the petals are group-specific unique ASVs.The diagram summarizes ASV counts across groups to illustrate shared or unique bacterial species characteristics.Sub-figures (a)-(d) and (e)-(h) correspond to different time points,instar stages,and concentration combinations:(a) 7 d, 5th-instar: control, low-concentration, high-concentration; (b) 7 d, 6th-instar: control, low-concentration, high-concentration; (c) 21 d, 5th-instar: control, low-concentration, high-concentration; (d) 21 d, 6th-instar: control, low-concentration, high-concentration; (e)7 d, 5th- and 6th-instar: control, low-concentration, high-concentration; (f) 21 d, 5th- and 6th-instar: control, low-concentration, high-concentration; (g) 7 d and 21 d, 5th-instar: control, low-concentration, high-concentration; (h) 7 d and 21 d, 6th-instar: control, low-concentration, high-concentration.

Figure S5

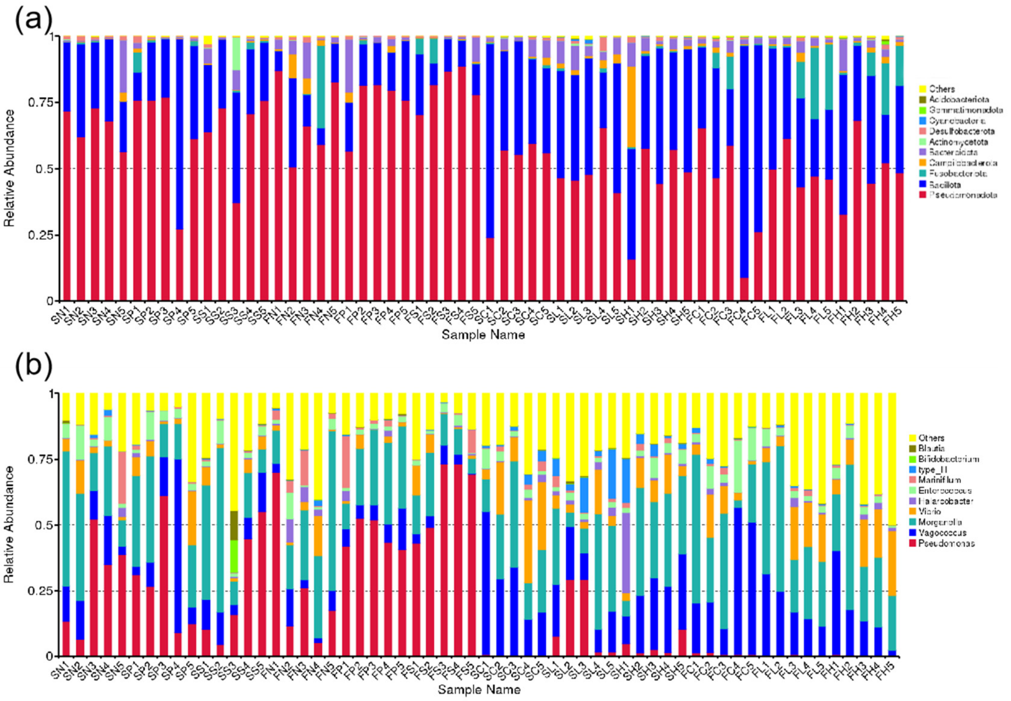

Figure S5 Cumulative histogram of relative abundance. (a) At the phylum level; (b) At the genus level.

Figure S6

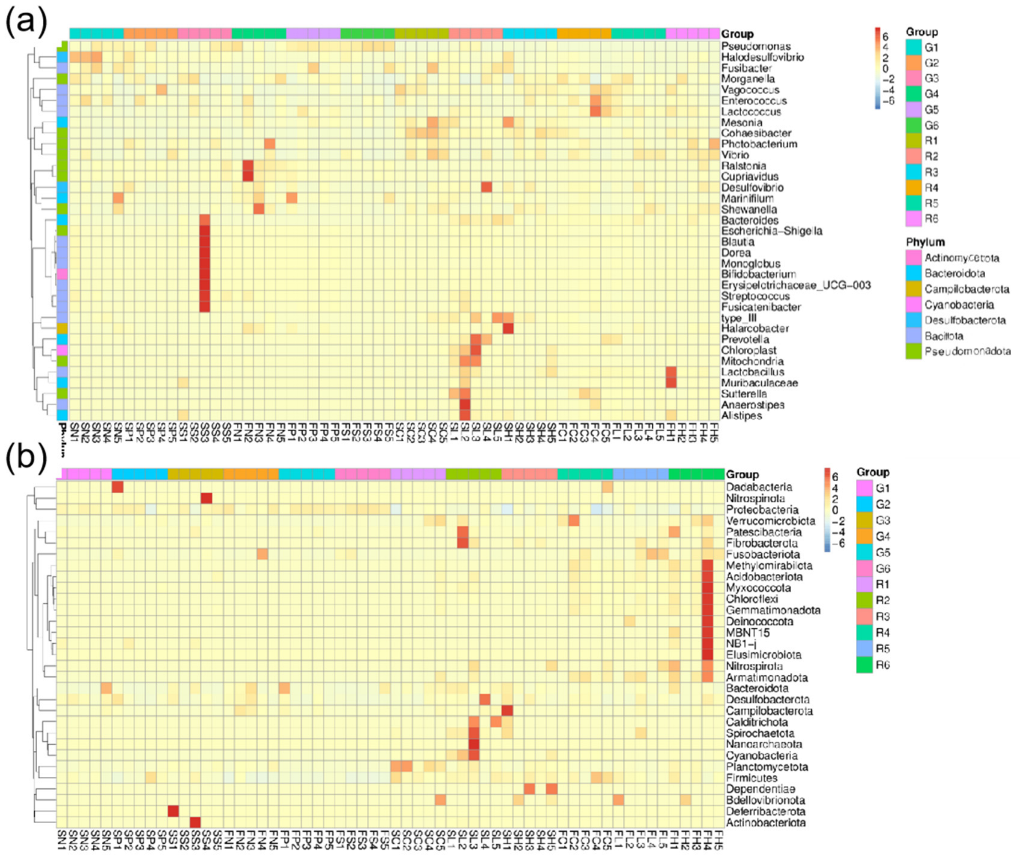

Figure S6 Abundance heatmaps of the top 35 bacterial phyla or genera. Red indicates high abundance, blue indicates low abundance. (a) Phylum-level heatmap; (b) genus-level heatmap.

Figure S7

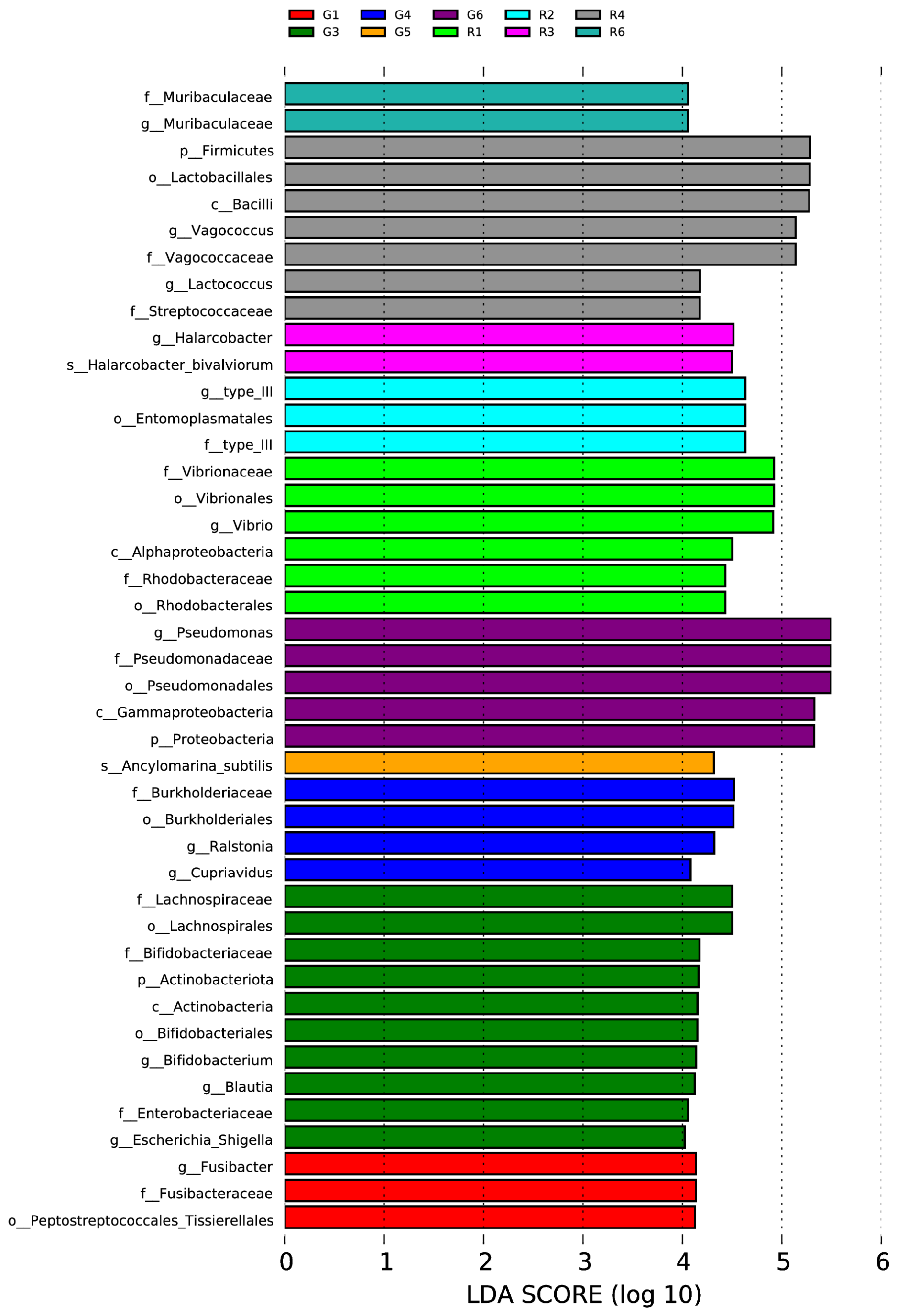

Figure S7 LDA score distribution histogram of biomarkers for the intestinal microbiota. Species with significant differences have LDA scores greater than the set threshold (default threshold: 4.0); the length of the histogram represents the LDA score, indicating the degree of influence of species with significant differences between groups.

Figure S8

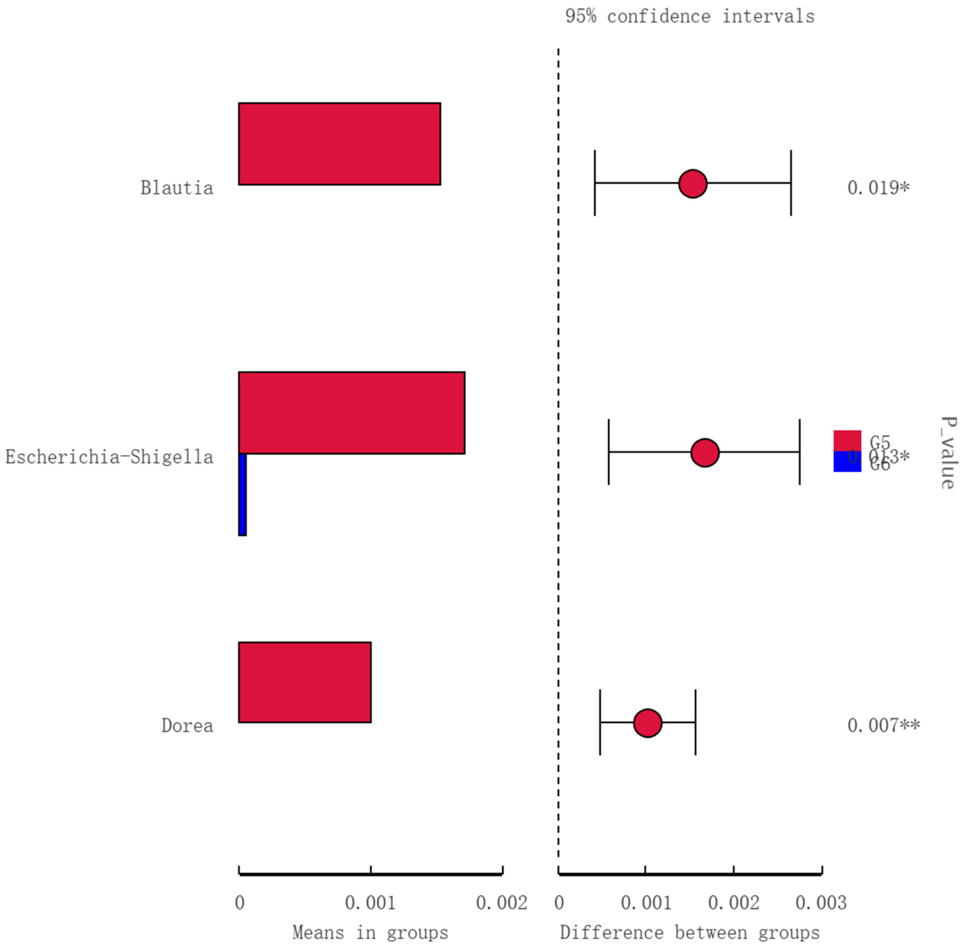

Figure S8 *T*-test analysis diagram (pertaining to classification). Displaying *T*-test results for taxa with significant differences between groups.

Figure S9

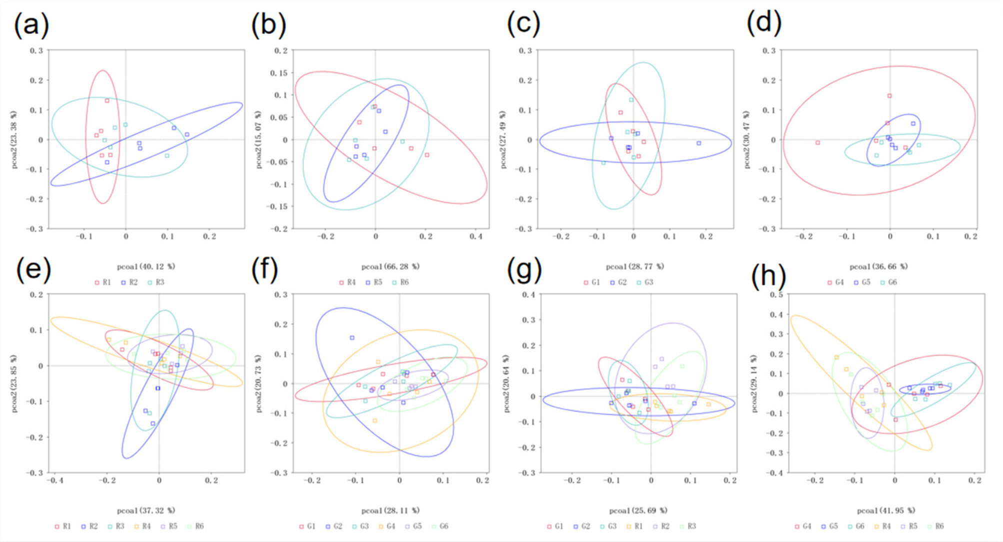

Figure S9 Principal component analysis (PCA)of gut microbiota in *Tachypleus tridentatus*. The x-and y-axes represent the first two principal components, with percentages indicating the contribution of each component to sample variance. Each dot represents one sample; samples within the same group are colored identically. Closer proximity indicates greater similarity in species composition, while wider separation denotes significant community differences. Sub-figures(a)–(h)correspond to the following group comparisons: (a)7 d, 6th-instar: control, low-concentration, high-concentration; (b) 7 d, 5th-instar: control, low-concentration, high-concentration; (c) 21 d, 6th-instar: control, low-concentration, high-concentration; (d) 21 d, 5th-instar: control, low-concentration, high-concentration; (e) 7 d, 5th- and 6th-instar: control, low-concentration, high-concentration; (f) 21 d, 5th- and 6th-instar: control, low-concentration, high-concentration; (g) 7 d and 21 d, 6th-instar: control, low-concentration, high-concentration; (h) 7 d and 21 d, 5th-instar: control, low-concentration, high-concentration.

Table S1

Table S1 Summary of three-way ANOVA results for physiological parameters in juvenile *Tachypleus tridentatus* exposed to PS-MPs

Source	df	CAT	SOD	MDA	LZM
Concentration (C)	2	*F*=1.938<br>*p* =0.167	*F*=50.530<br>***p* <0.001**	*F*=15.854<br>***p* <0.001**	F=1.801<br>*p* =0.187
Time(T)	1	*F*=14.635<br>***p* =0.001**	*F*=55.160<br>***p* <0.001**	*F*=18.569<br>***p* <0.001**	*F*=9.472<br>***p* =0.005**
Instar(I)	1	*F*=30.264<br>***p* <0.001**	*F*=7.624<br>***p* =0.009**	*F*=8.718<br>***p* =0.006**	*F*=1.656<br>*p* =0.210
C×T	2	*F*=1.068<br>*p* =0.360	*F*=37.827<br>***p* <0.001**	*F*=12.186<br>***p* <0.001**	*F*=1.758<br>*p* =0.194
C×I	2	*F*=1.541<br>*p* =0.236	*F*=30.456<br>***p* <0.001**	*F*=4.949<br>***p* =0.014**	*F*=0.196<br>*p* =0.823
T×I	1	*F*=1.511<br>*p* =0.231	*F*=43.944<br>***p* <0.001**	*F*=9.956<br>***p* =0.004**	*F*=3.000<br>*p* =0.096
C×T×I	2	*F*=0.470<br>*p* =0.631	*F*=20.902<br>***p* <0.001**	*F*=6.104<br>***p* =0.006**	*F*=0.024<br>*p* =0.976

Three-way ANOVA was performed with concentration (0, 10^2, 10^4 particles/L), time (7 d, 21 d), and instar (5th, 6th) as fixed factors, including all main effects and interaction terms. Values are presented as *F* (degrees of freedom) and *p*-values. Significant *p*-values (< 0.05) are indicated in bold. CAT: catalase; SOD: superoxide dismutase; MDA: malondialdehyde; LZM: lysozyme.
